# 

**Published:** 1908-09

**Authors:** 


### BOOKS RECEIVED

THE PRINCIPLES OF PATHOLOGY. By J. George Adair, M. A., M. D., L. L. D., F. R. S. Professor of Pathology in McGill Uniersity, and Pathologist to the Royal Victoria Hospital, Montreal, Late Fellow of Jesus College, Cambridge, England. Volume I, General Pathology, with 322 Engravings and 16 Plates. Lea & Febiger, Philadelphia.

ANATOMY, DESCRIPTIVE AND SURGICAL. By Henry Gray, F. R. S., Fellow of the Royal College of Surgeons; Lecturer on Anatomy at St. George's Hospital Medical School, London, England. Seventh Edition, Thoroughly Revised and Re-Edited with Additions by John Chalmers DaCosta, M. D., and Edward A. Spitzka, M. D. Illustrated with 1149 Engravings. Lea & Febiger, Philadelphia.

FOURTH ANNUAL REPORT of the Henry Phipps Institute for the Study, Treatment and Prevention of Tuberculosis, Edited by Joseph Walsh, A. M., M. D., 238 Pine Street, Philadelphia.

GOLDEN RULE OF DIETETICS. By A. L. eBnedict, A. M., M. D., Buffalo. C. V. Moseby, Publishers, St. Louis, Mo.

DISEASES OF THE SKIN. Ready Reference Hand Book. By George Thomas Jackson, M. D., with 99 Illustrations and 4 Plates. Sixth Edition, Thoroughly Revised. Lea & Febiger, Philadelphia.

BULLETIN OF THE BUREAU OF LABOR. March, 1908. Government Printing Office, Washington, D. C.

THE TRUE WAY OF LIFE. By Nanny Randolph Ball Baughman, Burlington, Iowa. Published by the Author.

INDUSTRIAL AND PERSONAL HYGIENE. By George M. Kober, M. D., L. L. D. A Report of the Committee or Social Betterment of Homes. Published by The President's Home Cimmission, Washington, D. C.

CONTRIBUTIONS TO THE SCIENCE OF MEDICINE AND SURGERY. By the Faculty of the New York Post-Graduate Medical School and Hospital in the Celebration of the Twenty-fifth Anniversary, 1908.

